# Supplementary figures and images for: Metabolism of long-chain fatty acids affects disulfide bond formation in Escherichia coli and activates envelope stress response pathways as a combat strategy
Source: PLoS Genet. 2020 Oct 20;16(10):e1009081. doi: 10.1371/journal.pgen.1009081 (PMC7598926; doi:10.1371/journal.pgen.1009081)

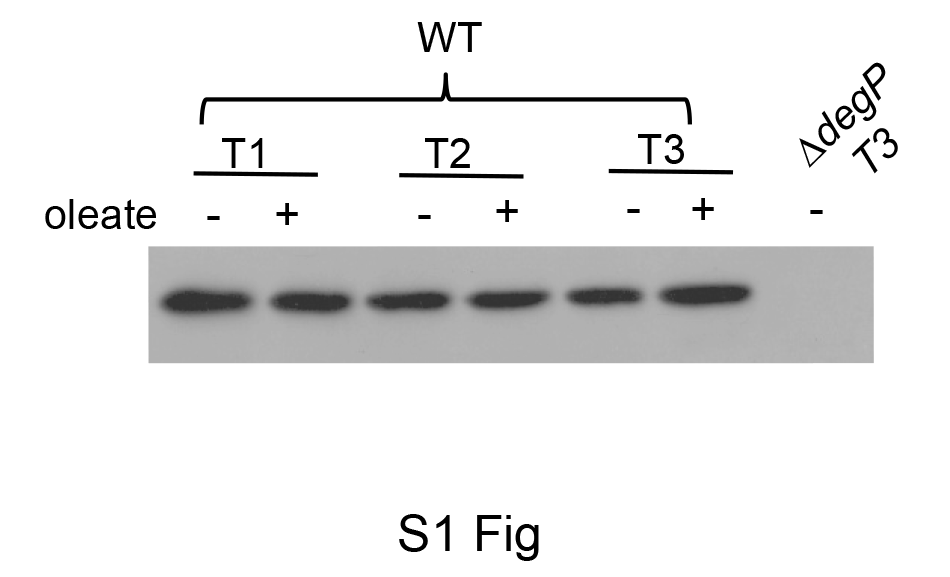

Supplement: S1 Fig — WT BW25113 was grown either in TBK-Brij or TBK-Ole, and cultures were harvested at time points, T1, T2, and T3 (indicated in Fig 1B). Lysates were prepared, samples were run on SDS-PAGE, and processed for Western blotting using an anti-DegP antibody. The band corresponding to DegP is shown (Mol. wt. ~50 kDa). (TIF) [file pgen.1009081.s001.tif]

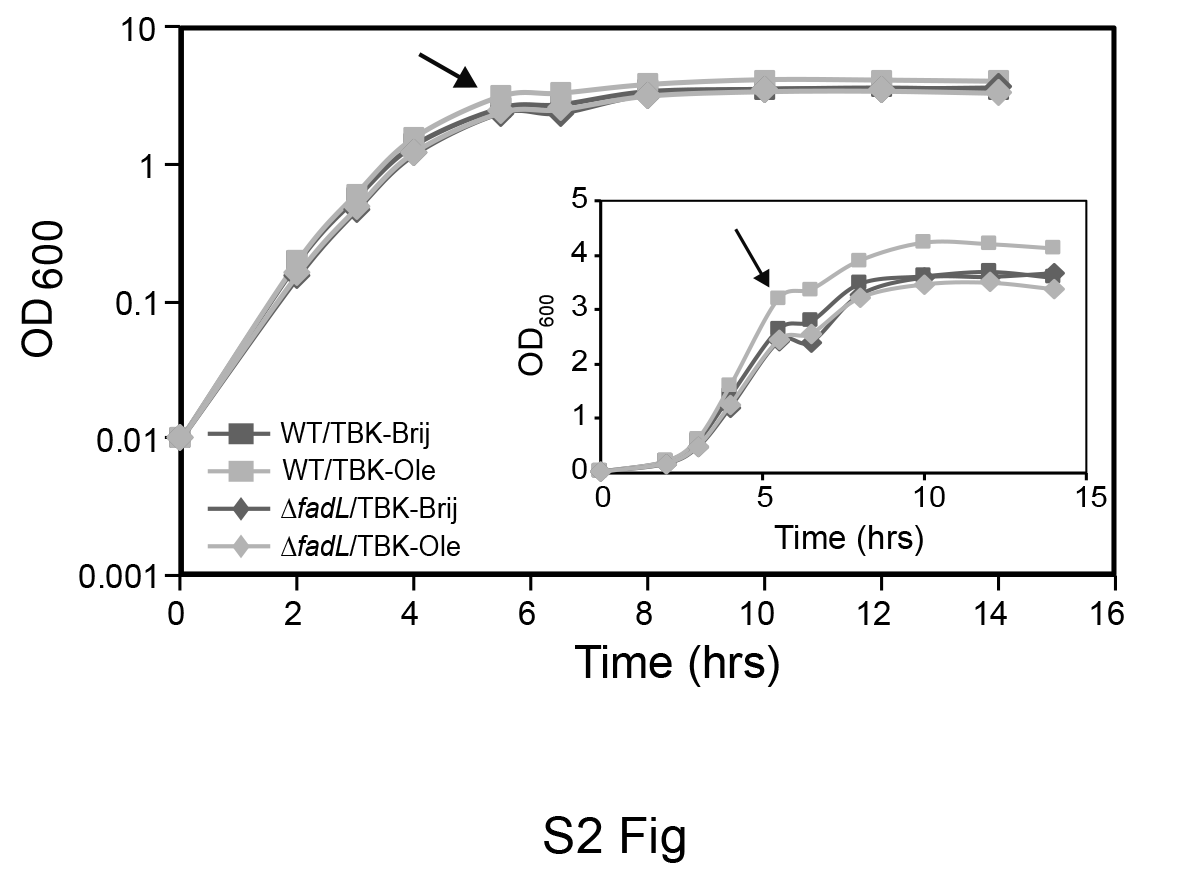

Supplement: S2 Fig — WT and ΔfadL strains were grown either in TBK-Brij or TBK-Ole. OD600 of the cultures was measured, and growth curves were plotted on a semi-logarithmic scale. The experiment was done two times. A representative dataset is shown. Arrow indicates time point T3 where cultures were harvested to check the redox state of DegP and DsbA in Figs 2F and 5B, respectively. Inset: The above growth curves were also plotted on a linear scale. (TIF) [file pgen.1009081.s002.tif]

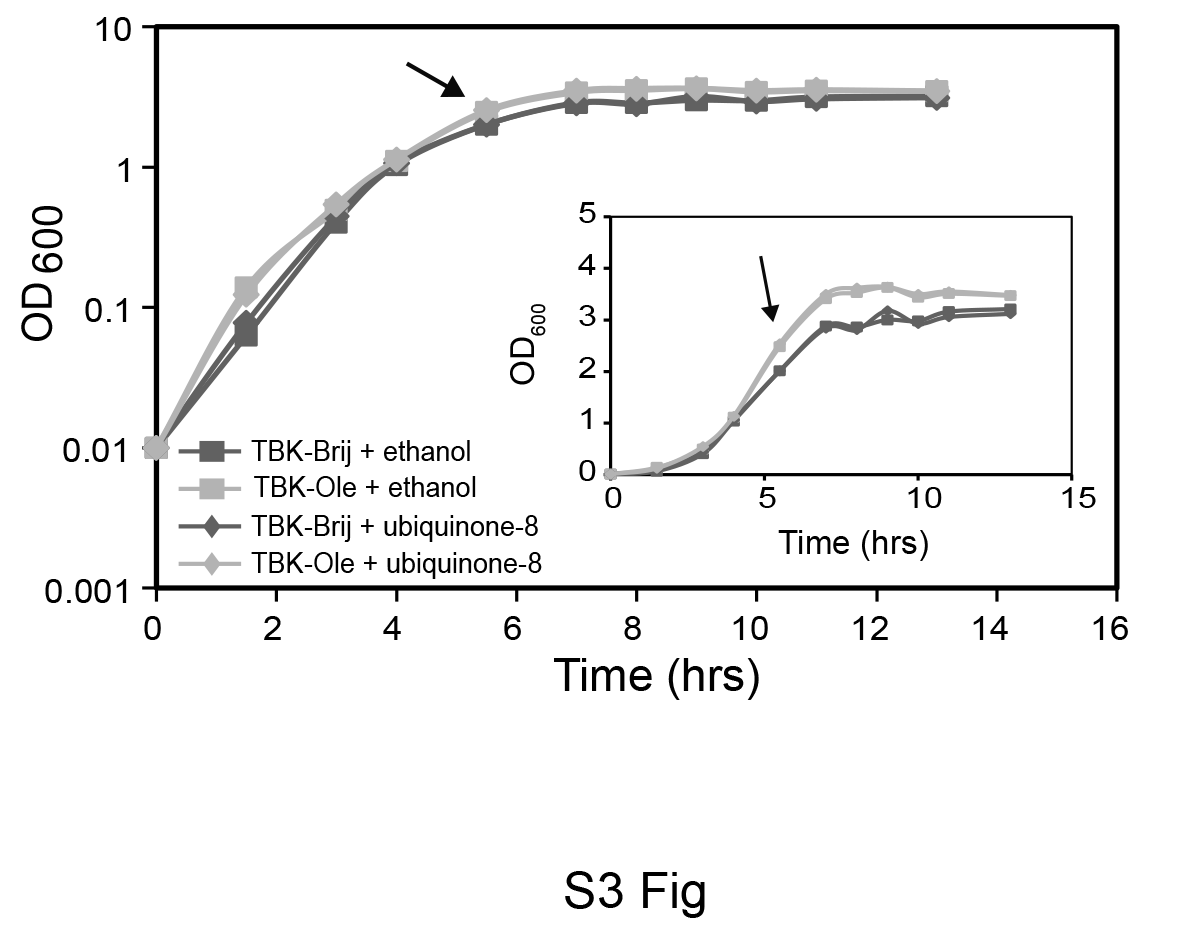

Supplement: S3 Fig — WT was grown in TBK-Brij and TBK-Ole supplemented either with 20 μM ubiquinone-8 or 0.1% ethanol. OD600 of the cultures was measured, and growth curves were plotted on a semi-logarithmic scale. The experiment was done two times. A representative dataset is shown. Arrow indicates time point T3, where cultures were harvested to check the redox state of DsbA in Fig 5C. Inset: The above growth curves were also plotted on a linear scale. (TIF) [file pgen.1009081.s003.tif]

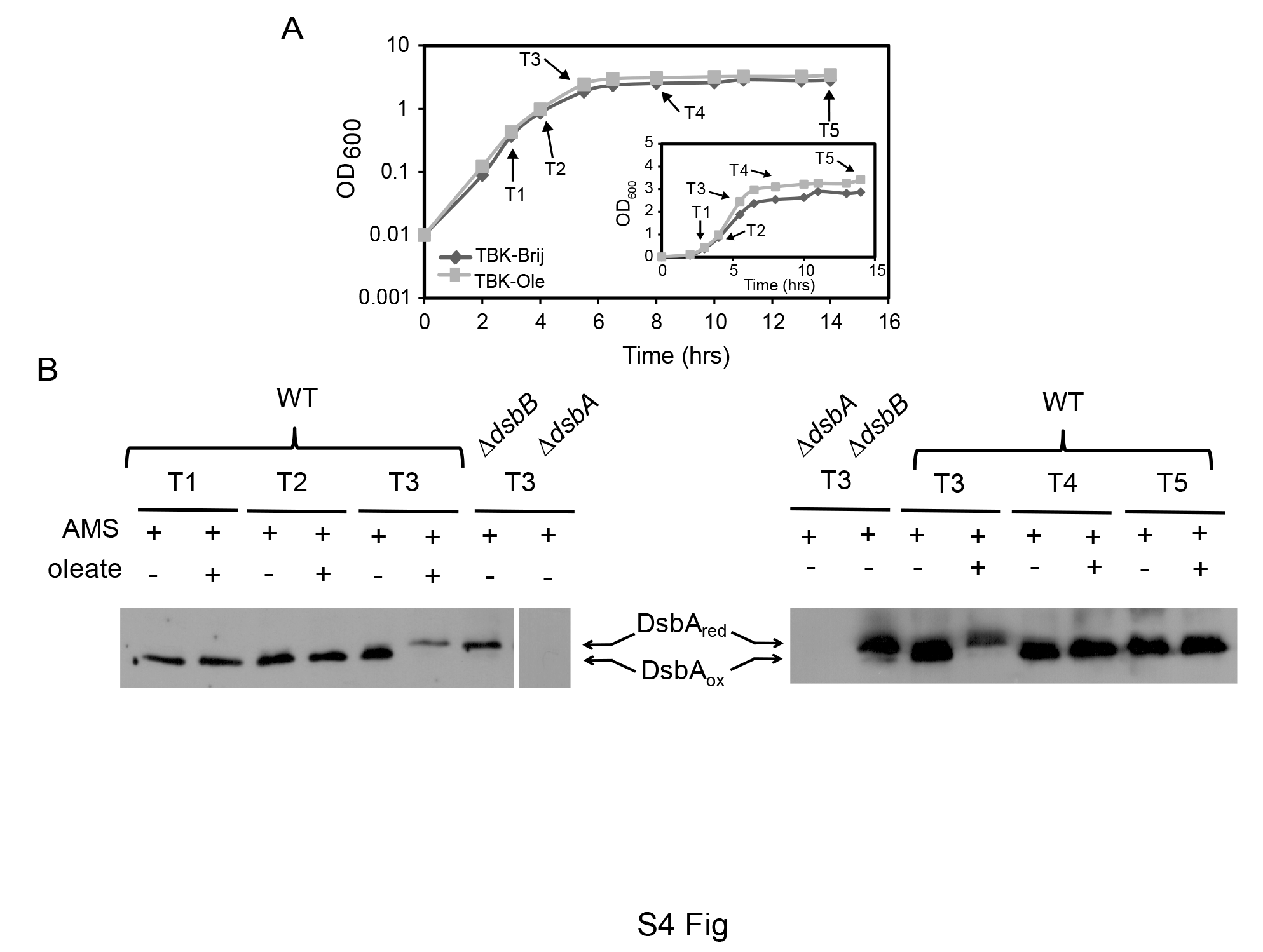

Supplement: S4 Fig — (A) Growth curve of WT in TBK-Brij and TBK-Ole. WT was grown either in TBK-Brij or TBK-Ole. OD600 of the cultures was measured, and growth curves were plotted on a semi-logarithmic scale. The experiment was done two times. A representative dataset is shown. T1, T2, T3, T4, and T5 indicate time points where cultures were harvested for determining the redox state of DsbA. Inset: The above growth curves were also plotted on a linear scale. (B) Redox state of DsbA in MG1655 at different phases of growth. WT was grown either in TBK-Brij or TBK-Ole. Cultures were harvested at different time points as indicated in S4A Fig and processed, as mentioned in the legend to Fig 5A. ΔdsbA and ΔdsbB cultured in TBK-Brij served as controls. DsbAox and DsbAred indicate oxidized and reduced forms of DsbA, respectively. (TIF) [file pgen.1009081.s004.tif]

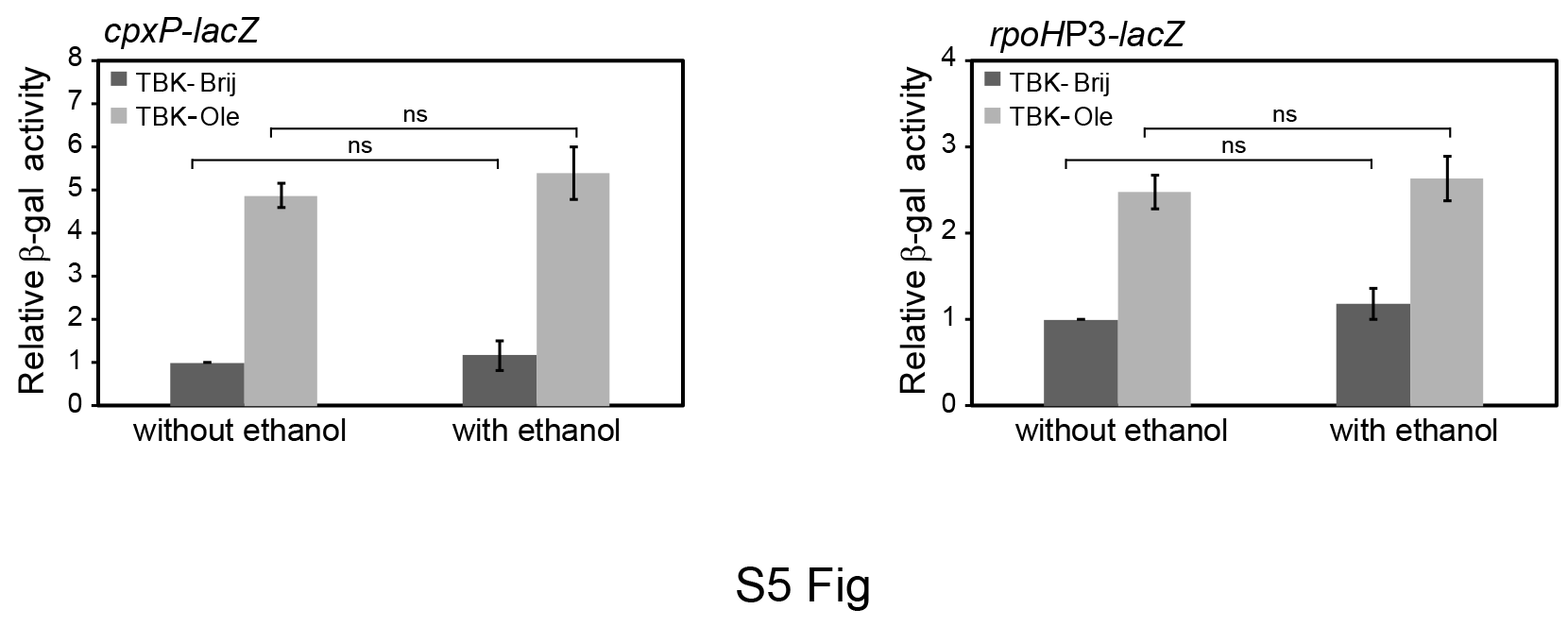

Supplement: S5 Fig — WT carrying either cpxP-lacZ (left panel) or rpoHP3-lacZ (right panel) reporter fusion was grown either in TBK-Brij or TBK-Ole with or without 0.1% ethanol supplementation. Cultures were harvested in the stationary phase (time point T5, as indicated in Fig 6A), and β-gal activity was measured. Data were normalized to the β-gal activity of WT in TBK-Brij without ethanol supplementation and represent the average (± S.D.) of three independent experiments. The average β-gal activity of the cpxP-lacZ reporter strain in TBK-Brij without ethanol supplementation was 34 (± 4) Miller units and that of rpoHP3-lacZ was 45 (± 9) Miller units. The p-values were calculated using the unpaired two-tailed Student’s t test (ns, P>0.03). (TIF) [file pgen.1009081.s005.tif]

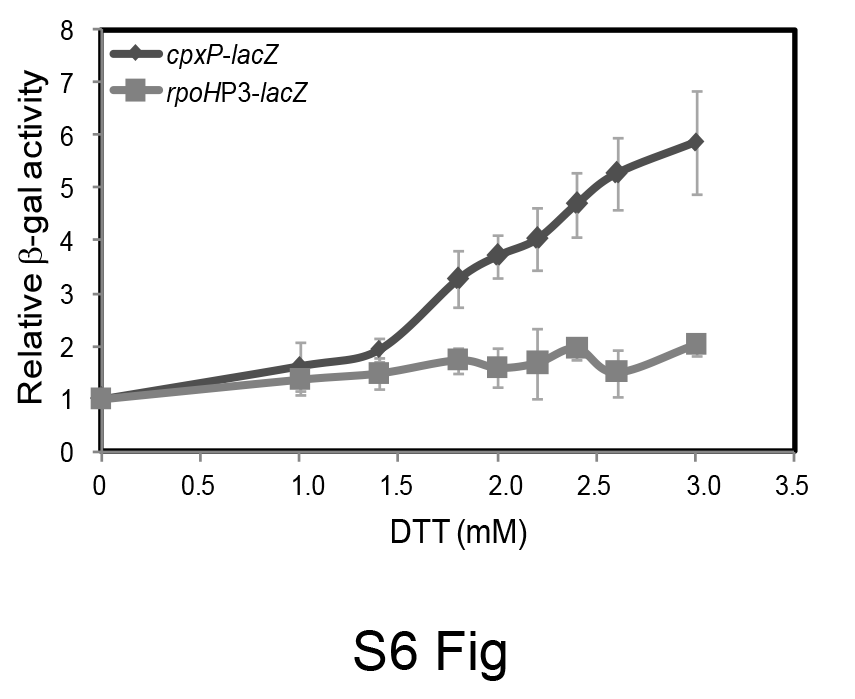

Supplement: S6 Fig — WT carrying either cpxP-lacZ or rpoHP3-lacZ reporter fusion was grown in TBK-Brij supplemented with increasing concentrations of DTT, as indicated. Cultures were harvested in the exponential phase, and β-gal activity was measured. Data were normalized to the β-gal activity of WT in TBK-Brij without DTT and represent the average (± S.D.) of three independent experiments. The average β-gal activity of the cpxP-lacZ reporter strain in TBK-Brij without DTT was 13 (± 5) Miller units and that of rpoHP3-lacZ was 45 (± 17) Miller units. (TIF) [file pgen.1009081.s006.tif]

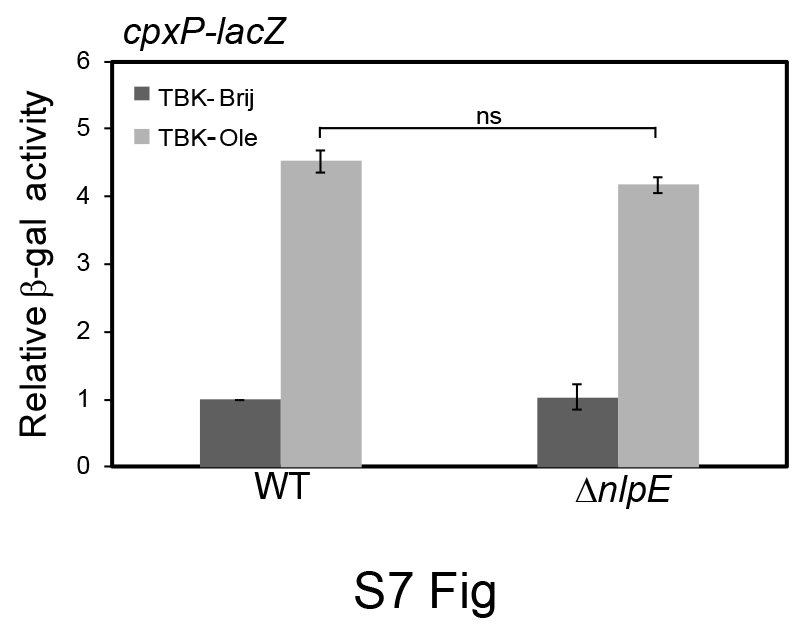

Supplement: S7 Fig — WT and ΔnlpE strains carrying cpxP-lacZ reporter fusion were grown either in TBK-Brij or TBK-Ole. Cultures were harvested in the stationary phase (time point T5, as indicated in Fig 6A), and β-gal activity was measured. Data were normalized to the β-gal activity of WT in TBK-Brij and represent the average (± S.D.) of three independent experiments. The average β-gal activity of the WT cpxP-lacZ reporter strain in TBK-Brij was 28 (± 5) Miller units. The p-values were calculated using the unpaired two-tailed Student’s t test (ns, P>0.03). (TIF) [file pgen.1009081.s007.tif]
