# Supplementary material for: Metabolism of long-chain fatty acids affects disulfide bond formation in Escherichia coli and activates envelope stress response pathways as a combat strategy
Source: PLoS Genet. 2020 Oct 20;16(10):e1009081. doi: 10.1371/journal.pgen.1009081 (PMC7598926; doi:10.1371/journal.pgen.1009081)
Supplement: S1 Table — (PDF) [file pgen.1009081.s008.pdf]

S1 Table. Strains and plasmids used in this study

Strain/Plasmid	Relevant genotype	Source [reference]
**Strains**		
BW25113	F⁻ Δ(*araD-araB*)*567* Δ*lacZ4787*(::rrnB-3) λ⁻ *rph-1* Δ(*rhaD-rhaB*)*568 hsdR514*	*E. coli* Genetic Stock Center
BW25142	*lacI^q^ rrnB3* Δ*lacZ4787 hsdR514* Δ(*araBAD*)*567* Δ(*rhaBAD*)*568* Δ*phoBR580 rph-1 galU95* Δ*endA9 uidA*(Δ*MluI*)::*pir-116 recA1*	Rao lab [1]
MG1655	F⁻ λ⁻ *rph-1*	*E. coli* Genetic Stock Center
MG1655 Δ*lac*	MG1655 Δ*lacX74*	Gross lab
AM1247	MG1655 *lacIZYA*::*frt nadA*::*Tn10 att*λ [P_{pspA}-*lacZ*], Tet^r	Reddy Lab [2]
CAG45114	MG1655 Δ*lacX74* λ[P_{rpoHP3}-*lacZ*]	Gross Lab [3]
CAG53524	MC1061 [ϕλ*rpoH*P3::*lacZ*] *degS*ΔPDZ::*kan*, Kan^r	Gross lab [4]
CAG55907	CAG45114 Δ*relA* Δ*spoT*::*cam*, Cam^r	Gross lab [5]
DH300	MG1655 Δ(*argF-lac*)*U169* [$P_{rprA142}$-*lacZ*]	Ades Lab [6]
RI89	F⁻ Δ*araBAD714* (*araD139*)$_{B/r}$ Δ(*codB-lacI*)*3 phoR82 galK16 galE15* λ⁻ *e14⁻ relA1 rpsL150 spoT1 mcrB1*	Sardesai lab [7]
SEA4166	MG1655 Δ*lacX74* λ*RS88* [P_{cpxP}-*lacZ*]	Ades Lab
SEA4170	MG1655 Δ*lacX74* λ*RS88* [P_{spy}-*lacZ*]	Ades Lab
BW25113 Δ*fadL*	BW25113 *fadL::kan*, Kan^r	Keio collection [8]
BW25113 Δ*nuoK*	BW25113 *nuoK::kan*, Kan^r	Keio collection [8]
BW25113 Δ*sdhB*	BW25113 *sdhB::kan*, Kan^r	Keio collection [8]
BW25113 Δ*degP*	BW25113 *degP::kan*, Kan^r	Keio collection [8]
BW25113 Δ*dsbA*	BW25113 *dsbA::kan*, Kan^r	Keio collection [8]
BW25113 Δ*dsbB*	BW25113 *dsbB::kan*, Kan^r	Keio collection [8]
BW25113 Δ*cpxR*	BW25113 *cpxR::kan*, Kan^r	Keio collection [8]
RC15082	BW25113 *att*λ::[Kan P_{fadE} -*lacZ oriR6K*], Kan^r	This work
RC5264	P1 (BW25113 *fadL*::*kan*) X RI89, Kan^r	This work
RC15062	P1 (BW25113 *fadE*::*kan*) X RI89, Kan^r	This work

RC5327	P1 (BW25113 *dsbA*::*kan*) X RI89, Kan^r	This work
RC5328	P1 (BW25113 *dsbB*::*kan*) X RI89, Kan^r	This work
RC5337	P1 (BW25113 *dsbA*::*kan*) X MG1655, Kan^r	This work
RC5338	P1 (BW25113 *dsbB*::*kan*) X MG1655, Kan^r	This work
RC15106	P1 (AM1247) X MG1655 Δ*lacX74 nadA*$^+$	This work
RC15006	P1 (BW25113 *fadL*::*kan*) X CAG45114, Kan^r	This work
RC15005	P1 (BW25113 *fadE*::*kan*) X CAG45114, Kan^r	This work
RC5317	P1 (CAG53524) X CAG45114, Kan^r	This work
RC15104	P1 (BW25113 *rseB*::*kan*) X CAG45114, Kan^r	This work
RC15103	RC15104, *kan* cassette flipped out	This work
RC15105	P1 (BW25113 *relA*::*kan*) X CAG45114, Kan^r	This work
RC15095	P1 (BW25113 *cydD*::*kan*) X CAG45114, Kan^r	This work
RC15020	P1 (BW25113 *fadL*::*kan*) X SEA4166, Kan^r	This work
RC15019	P1 (BW25113 *fadE*::*kan*) X SEA4166, Kan^r	This work
RC15043	P1 (BW25113 *ackA*::*kan*) X SEA4166, Kan^r	This work
RC5320	RC15043, *kan* cassette flipped out	This work
RC5322	P1 (BW25113 *pta*::*kan*) X RC5320, Kan^r	This work
RC5302	P1 (BW25113 *cpxA*::*kan*) X SEA4166, Kan^r	This work
RC15096	P1 (BW25113 *cydD*::*kan*) X SEA4166, Kan^r	This work
RC18029	P1 (BW25113 *nlpE*::*kan*) X SEA4166, Kan^r	This work
**Plasmids**		
pCP20	pSC101 *ori cI857* λ-P$_R$ *flp* ts Amp^r Cam^r	[9]
pINT-ts	*oriR6K int* Amp^r	Rao lab [1]
pAH125	*oriR6K,* MCS-*lacZ t0 att*λ Kan^r	Rao lab [1]
pMS02	*oriR6K,* MCS P$_{fadE}$-*lacZ t0 att*λ Kan^r	This work

References

1. Haldimann A, Wanner BL. Conditional-replication, integration, excision, and retrieval plasmid-host systems for gene structure-function studies of bacteria. Journal of bacteriology. 2001;183(21):6384-93. Epub 2001/10/10.

2. Mychack A, Amrutha RN, Chung C, Cardenas Arevalo K, Reddy M, Janakiraman A. A synergistic role for two predicted inner membrane proteins of *Escherichia coli* in cell envelope integrity. Molecular microbiology. 2019;111(2):317-37. Epub 2018/10/29.
3. Ades SE, Grigorova IL, Gross CA. Regulation of the alternative sigma factor σ^E during initiation, adaptation, and shutoff of the extracytoplasmic heat shock response in *Escherichia coli*. Journal of bacteriology. 2003;185(8):2512-9. Epub 2003/04/03.
4. Chaba R, Alba BM, Guo MS, Sohn J, Ahuja N, Sauer RT, et al. Signal integration by DegS and RseB governs the σ^E-mediated envelope stress response in *Escherichia coli*. Proceedings of the National Academy of Sciences of the United States of America. 2011;108(5):2106-11. Epub 2011/01/20.
5. Mutalik VK, Nonaka G, Ades SE, Rhodius VA, Gross CA. Promoter strength properties of the complete sigma E regulon of *Escherichia coli* and *Salmonella enterica*. Journal of bacteriology. 2009;191(23):7279-87. Epub 2009/09/29.
6. Majdalani N, Hernandez D, Gottesman S. Regulation and mode of action of the second small RNA activator of RpoS translation, RprA. Molecular microbiology. 2002;46(3):813-26. Epub 2002/11/02.
7. Rietsch A, Belin D, Martin N, Beckwith J. An *in vivo* pathway for disulfide bond isomerization in *Escherichia coli*. Proceedings of the National Academy of Sciences of the United States of America. 1996;93(23):13048-53. Epub 1996/11/12.
8. Baba T, Ara T, Hasegawa M, Takai Y, Okumura Y, Baba M, et al. Construction of *Escherichia coli* K-12 in-frame, single-gene knockout mutants: the Keio collection. Molecular systems biology. 2006;2:2006.0008. Epub 2006/06/02.
9. Datsenko KA, Wanner BL. One-step inactivation of chromosomal genes in *Escherichia coli* K-12 using PCR products. Proceedings of the National Academy of Sciences of the United States of America. 2000;97(12):6640-5. Epub 2000/06/01.
