# Supplementary material for: Metabolism of long-chain fatty acids affects disulfide bond formation in Escherichia coli and activates envelope stress response pathways as a combat strategy
Source: PLoS Genet. 2020 Oct 20;16(10):e1009081. doi: 10.1371/journal.pgen.1009081 (PMC7598926; doi:10.1371/journal.pgen.1009081)
Supplement: S2 Table — (PDF) [file pgen.1009081.s009.pdf]

S2 Table. Primers used in this study

Primer Name	Purpose	Sequence (5'-3')
**Primers used for cloning *fadE* cis element in pAH125 and its verification**		
MS43	Forward primer for cloning *fadE* promoter in pAH125	ACCGGGTACCATTGATTTAAGAATTTTCAGGTCGGATGC
MS44	Reverse primer for cloning *fadE* promoter in pAH125	CGTGAATTCCCGTAGCGAGAATACTCAAAATCATCAT
BS106	Sequencing/verification primer for cloning in pAH125	TTGTCGGTGAACGCTCTCCT [1]
MS49	Sequencing/verification primer for cloning in pAH125	TAAAACGACGGCCAGTGAATCC
Restriction sites are underlined		
**Primers used for strain verification of single integrants at the *att*λ site**		
GA22	Forward primer specific to *att*λ site on *E. coli* chromosome for confirmation of single-copy integration of reporter plasmids	GGCATCACGGCAATATAC [1]
GA23	Forward primer specific to pAH125 to identify multiple-copy integration of reporter plasmids	ACTTAACGGCTGACATGG [1]
GA25	Reverse primer specific to *att*λ site on *E. coli* chromosome for confirmation of single-copy integration of reporter plasmids	TCTGGTCTGGTAGCAATG [1]
GA29	Reverse primer specific to pAH125 to identify multiple-copy integration of reporter plasmids	TGCGAGGCTTTGTGCTTC [1]

References

1. Singh B, Arya G, Kundu N, Sangwan A, Nongthombam S, Chaba R. Molecular and Functional Insights into the Regulation of D-Galactonate Metabolism by the Transcriptional Regulator DgoR in *Escherichia coli*. Journal of bacteriology. 2019;201(4):e00281-18. Epub 2018/11/21.
